# COVERater—A Free Application for Training Researchers to Accurately Estimate Species Cover in Terrestrial and Aquatic Ecosystems

**DOI:** 10.1002/ece3.70447

**Published:** 2024-10-16

**Authors:** Madelon M. Cruickshank, Angela T. Moles, Samuel A. Debono, Zoe A. Xirocostas

**Affiliations:** ^1^ Evolution & Ecology Research Centre, School of Biological, Earth and Environmental Sciences UNSW Sydney Sydney New South Wales Australia; ^2^ Pegleg Software (Peglegsoftware.com.au) Sydney New South Wales Australia; ^3^ School of Life Sciences University of Technology Sydney Sydney New South Wales Australia

**Keywords:** cover estimates, percentage cover, training tool, visual estimation

## Abstract

Visual estimates of cover are widely used among ecologists, from describing vegetation communities to tracking and monitoring species' abundance. However, despite the known bias associated with visual estimates, no standardised training is available to improve these measurements. We developed a free online training tool, the COVERater, that effectively teaches users to visually estimate the percent cover of species in a variety of ecosystems (including alpine heath, arid lands, coral reefs, temperate reefs and wetlands). Prior to training, users with prior professional experience estimated species cover to an average inaccuracy of 5.2%, while users with no experience estimated cover to an average inaccuracy of 7.6%. COVERater training took an average of 31 min and 68 images, and reduced the estimate inaccuracy of users with no prior experience to 5.2%. There was no significant loss of estimate accuracy over 100 days following training. The COVERater can be used anywhere in the world, by data collectors of all experience levels, for projects spanning all spatial scales. By providing researchers with standardised training, our application can reduce variation in cover estimates that arise from human biases, allowing for comparable estimates across global collaborative projects and data syntheses. We encourage all relevant scientists to include COVERater training in their protocols to quantify cover with greater accuracy, improve the veracity of their results and make better inferences about our biosphere.

## Graphical abstract

We present the COVERater, a free online training tool that reduces the inaccuracy of users' visual estimates of species cover. This application will help to standardise protocols and ensures comprehensive and reliable data collection from those with no prior estimating experience.

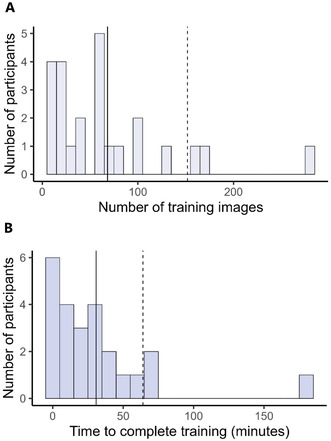

## 1 Introduction

Quantifying the per cent cover of species is important for describing vegetation communities (Symstad, Wienk, and Thorstenson 2008), determining habitat availability (Ocock et al. 2018), measuring ecosystem changes (Friedmann et al. 2011) and informing monitoring programmes (Brundrett et al. 2019; Helm and Mead 2004). There are multiple ways to measure per cent cover, and each method carries its own limitations and biases (Bergstedt, Westerberg, and Milberg 2009; Killourhy, Crane, and Stehman 2016). For example, remote sensing methods can provide accurate measurements of species cover, but their use is limited by the costs, expertise and time required to ground truth images for data calibration (Anderson and Gaston 2013; Berni et al. 2009). Some researchers capture images in the field for post‐processing with specialised software (e.g., ImageJ), however, the time taken to photograph, upload and process images may be unfeasible for those under time constraints or with large sample sizes (Ricotta et al. 2014). Most studies simply have experts make visual estimates, either of per cent cover of species in quadrats (Lawley et al. 2016), or by placing each species into cover classification categories such as Braun‐Blanquet (Bezuidenhout et al. 1994; Mirkin and Naumova 2009). Previous studies have called for the need to decrease the inaccuracy of cover estimates in both aquatic (Finn et al. 2010) and terrestrial contexts (Archaux et al. 2009). However, there are no accessible and standardised training materials available to help bring visual cover estimates closer to real‐world values. Here, we introduce the COVERater (www.coverater.com), a freely available online application that will improve the speed and accuracy in which cover estimates are made, and increase their comparability across studies spanning local to global scales.

The COVERater app (www.coverater.com) presents the user with an image of a real terrestrial vegetation or sessile marine community, and asks them to estimate the per cent cover of a selected species, indicating their estimate on a sliding scale from 0% to 100%. The user's answer is compared with the ‘true’ value, calculated manually using digital image analysis, and the user is given feedback on their estimate (e.g., ‘So close! Your answer was: X%, the cover estimate percentage is: X%’). The COVERater then presents the user with subsequent images, and training ceases when the user achieves a running average of 98% accuracy or higher on their 10 most recently estimated images. The COVERater is modelled off and builds on the ZAX Herbivory Trainer (Xirocostas et al. 2022), an app that trains observers to visually estimate the percentage of leaf area that has been damaged by herbivores.

We first aimed to test the hypothesis that using the COVERater will reduce users' inaccuracy when visually estimating per cent cover. There is currently no way to know whether current training methods are effective in decreasing levels of inaccuracy. This is important to consider as it determines whether training is worth the time, energy and organisation efforts. A study completed by Gallegos Torell and Glimskär (2009) had participants estimate 24 images created in Photoshop which mimicked vegetation. Within three sessions where feedback was received, participants could bring estimates in the field to within 3% inaccuracy (Gallegos Torell and Glimskär 2009). This study only explored a limited range of vegetation communities with photos manipulated in Photoshop (Adobe Inc.). However, this study still indicates the potential for feedback in the context of species cover to be a successful training technique for increasing accuracy levels.

Second, we asked how many images and how much time was required to reduce user inaccuracy down to 0%–2% using the COVERater. Existing methods of training can vary from half a day (Vittoz et al. 2010) to multi‐day in situ training (Finn et al. 2010), but based on similar approaches used in Xirocostas et al. (2022), we anticipate our training app will take much less time to complete.

Next, we asked how often individuals will need to retrain with the COVERater to maintain low levels of estimate inaccuracy. Reviewing and refining skills are commonly applied in various contexts, including exam preparation and education literature (Cross, Whitelock, and Mittelmeier 2016). Retraining exists in varying forms for cover estimates but is not always undertaken (Helm and Mead 2004), and it is often left to the observer to decide whether retraining is necessary (Finn et al. 2010; Gallegos Torell and Glimskär 2009). Determining how often people need to retrain will ensure estimates remain accurate and save time in the future by providing a clear recommendation for optimal accuracy retention.

People with experience conducting fieldwork and making cover estimates are often used as the ‘gold standard’ and often assumed to be highly accurate (Groom and Whild 2017). Experience in estimating cover can reduce overlooking and misidentification errors (Groom and Whild 2017; Killourhy, Crane, and Stehman 2016). However, even experienced and highly trained individuals can be susceptible to non‐sampling errors in their estimates. Some studies have compared the accuracy between and within participants (Archaux et al. 2006; Gorrod and Keith 2009), although they have rarely compared estimator accuracy to a ‘true value’. We hypothesise that individuals with professional experience in estimating cover will have a lower baseline estimate inaccuracy than participants with no prior estimation experience.

We hope the COVERater app will help to train students and increase the accuracy of cover estimates made by researchers in a range of ecosystems worldwide.

## 2 Materials and Methods

### 2.1 Data Collection

We began by obtaining images of different environments from the field, and from colleagues. Image locations span multiple continents including Europe, Asia and Australia. All images were taken from an aerial perspective, between 0.25 and 5 m from the surface, and included one or more sessile aquatic or terrestrial species.

Next, we calculated per cent cover for two focal species in each image. Focal species were selected by choosing the first identifiable species closest to the top, left‐hand corner and the first identifiable species closest to the centre of the image. If the same species occurred at both these points, the next closest species from the centre of the image was chosen. Where there was only one species present in the image, a species which is not present in the image, but from the same environment, was used as the second focal species with the percentage cover in the area being 0%. As of 10/11/2022 a total of 200 images are currently included in this application.

We used Photoshop version 22.5.9 (Adobe Inc.) to quantify the per cent cover of each focal species. Using the brush tool, we covered the area of each species in white on a separate layer with a black background. To reduce the risk of misidentification, a subset of 20 randomly sampled images were checked by another researcher post processing. We quantified the projected cover of the target species within each image using the auto‐threshold function from the black and white contrasted image using ImageJ (Schneider, Rasband, and Eliceiri 2012). The image database includes levels of species cover spanning 0%–100%.

To test the effectiveness of the COVERater app we recruited participants from the School of Biological, Earth and Environment Sciences at UNSW Sydney through email (with prior human ethics advisory panel approval HC220176 from UNSW Sydney). Our study included 24 participants with no prior professional experience in estimating species cover and 10 participants with professional experience in estimating species cover (i.e., a history of employment that included making cover estimates).

Two versions of the application were developed to allow participants to progress through the application smoothly. Each version of the app was password protected. The first version was created for participants without prior professional cover estimate experience. To quantify these users' baseline accuracy prior to training, participants were first shown an instruction page before estimating the per cent cover of species in 50 random images without feedback on their accuracy levels. Immediately after, these participants completed the training module where they visually estimated the percentage cover of the target species. Following each estimate, users received feedback indicating their accuracy which allowed them to improve with each successive image. Training was completed once participants achieved a running average of 98% accuracy or higher on their 10 most recently estimated images. An integrated database within the application recorded the number of images, length of time and average accuracy levels for each user. Then, participants immediately estimated the percentage cover of a focal species in 50 images without receiving feedback on the estimate accuracy.

To determine whether users' inaccuracy increased over time, participants were reminded to estimate percentage cover in 50 images without feedback at intervals including immediately after, 1 h, 1 day, 1 week, 1 month and 3 months after completing the online training module.

A second version of the application was made for participants with prior professional experience in estimating per cent cover. These experienced participants progressed through the application to estimate the percentage cover of species in 50 images without feedback. Data were collected to compare the accuracy of participants with professional experience in cover estimates against the accuracy of participants with no cover estimate experience.

### 2.2 Data Analysis

Statistical analyses were performed in RStudio version 4.2.0 (R Core Team 2021). All model assumptions were checked prior to analyses and were satisfied unless explicitly stated otherwise. Upon the completion of data collection, all data were removed from the integrated database in our web app and anonymised. Unless otherwise specified, analyses were performed on data collected from users with no prior experience in estimating per cent cover.

To test the efficacy of the COVERater, we performed a paired samples t‐test comparing users' average estimate inaccuracy on 50 random images without feedback, before and immediately after training.

To determine whether estimate inaccuracy increased with time since training, we performed a linear mixed‐effects model using the *lmer* function in the lmerTest package (Kuznetsova, Brockhoff, and Christensen 2017). Our response variable was users' average estimate inaccuracy, and the predictor variable was the number of days that had passed since initial training occurred with a random effect term included for each user.

To determine whether users with professional experience made more accurate cover estimates than users with no experience we performed an unpaired two‐samples Wilcoxon test on each group's average estimate accuracy prior to training. We used non‐parametric analysis as our data could not be transformed to fit assumptions of normality.

## 3 Results

**FIGURE 1:**
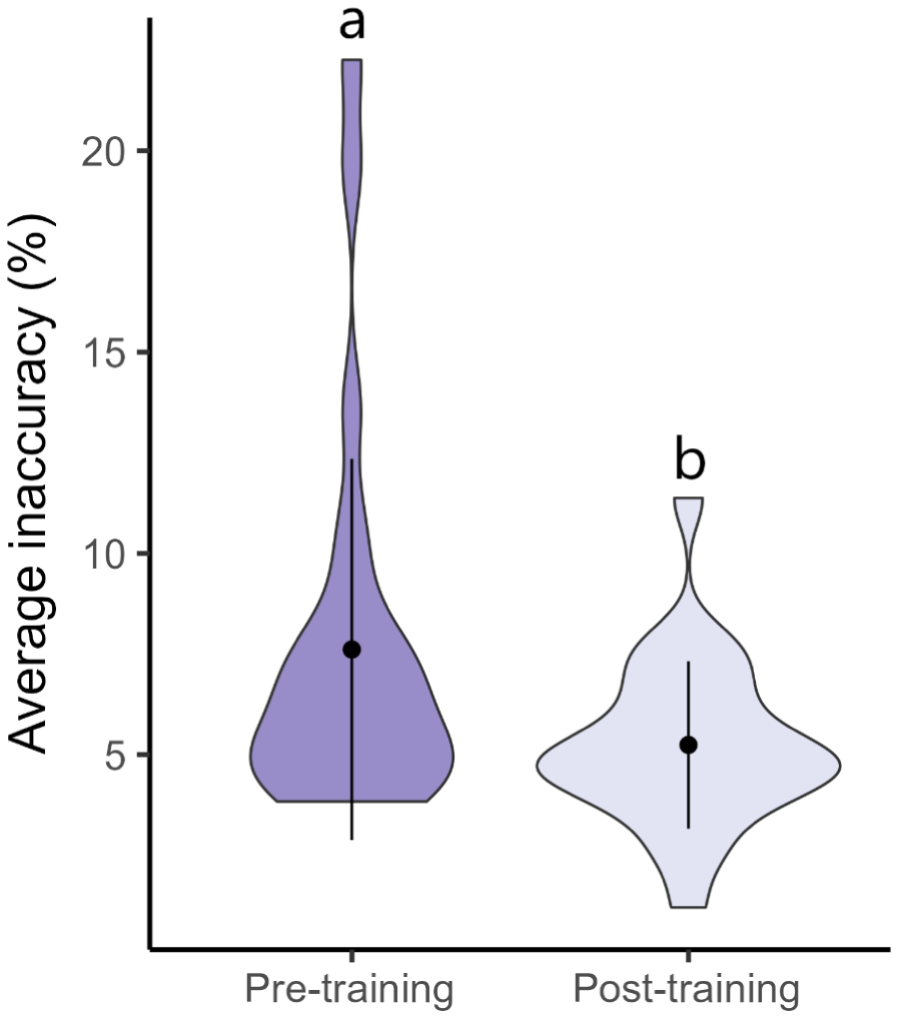
Violin plot of participants' average estimate inaccuracy before and after completing COVERater training. Points represent means and bars represent standard deviation. Different lowercase letters above plots denote significant differences between groups (*p* = 0.003).

The COVERater training application significantly reduces users' cover estimate inaccuracy (Figure 1; *p* = 0.003, *t* = −3.28, df = 23). Prior to training, users visually estimated per cent cover with an average inaccuracy of 7.6%, which was reduced to 5.2% upon completion of training.

**FIGURE 2:**
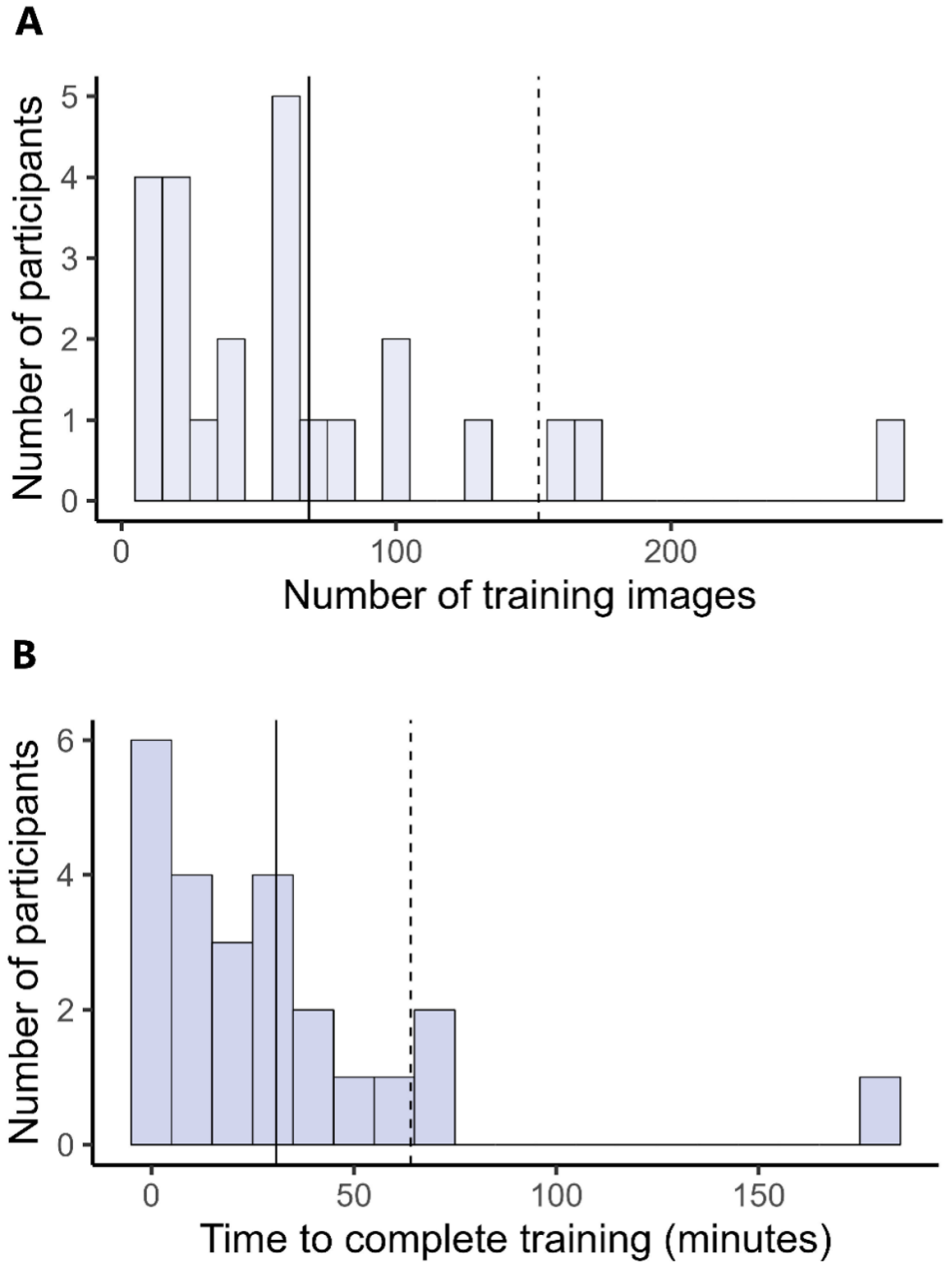
Histograms representing (A) the number of images and (B) the number of minutes users took to complete COVERater (*n* = 24). Solid lines represent means and dashed lines represent the 90th percentile. Bars are binned by intervals of 10.

To complete COVERater training, it takes users an average of 68 images (Figure 2A) and 31 min (Figure 2B). Ninety per cent of users reached an average inaccuracy of 2% or less on their last 10 cover estimates within 152 images and 64 min (Figure 2A,B).

**FIGURE 3:**
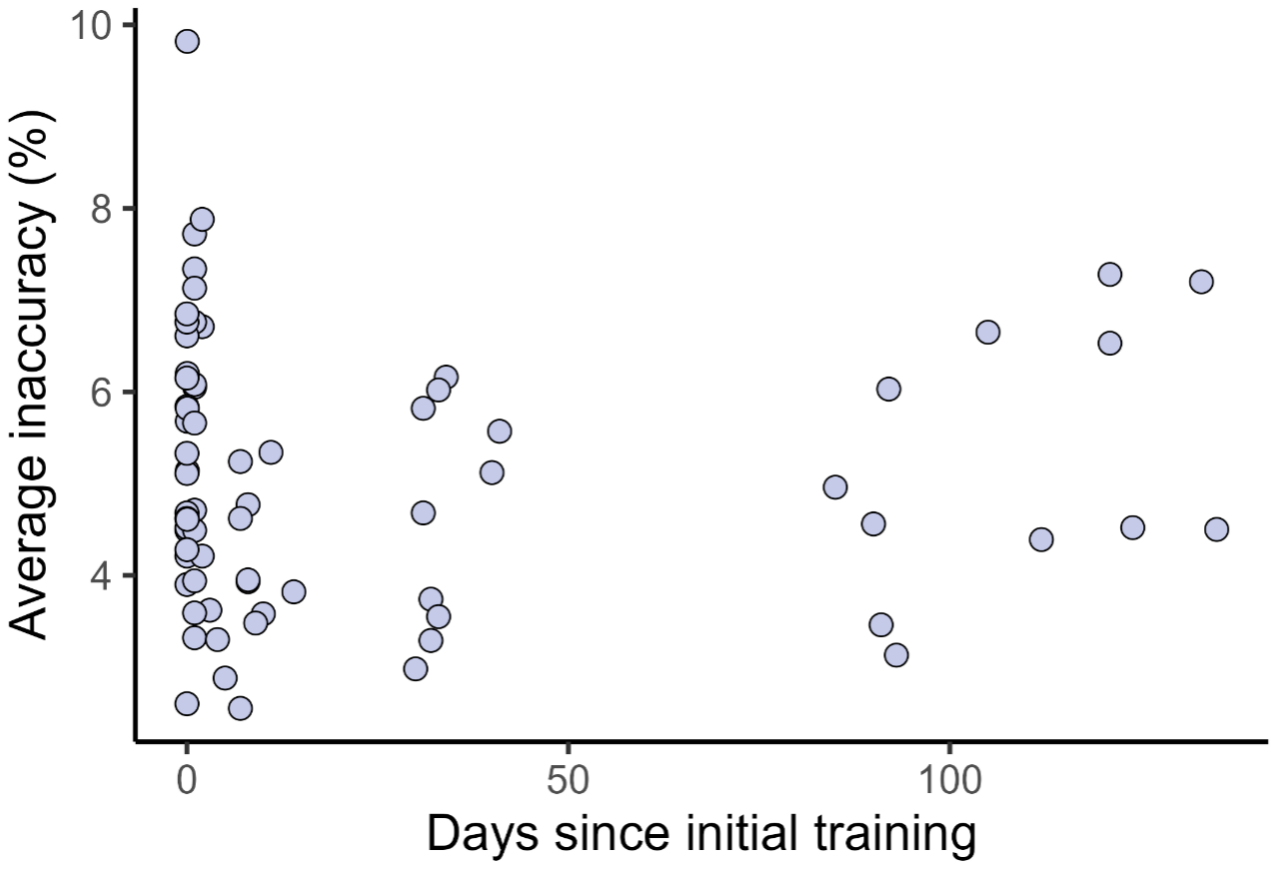
Users' average estimate inaccuracy was not associated with the amount of time that had passed since initial training (*p* = 0.83).

Participant inaccuracy remained low over time, with participants performing cover estimates with similar inaccuracy levels from 1 h, to 100 days after training (Figure 3; *p* = 0.83).

**FIGURE 4:**
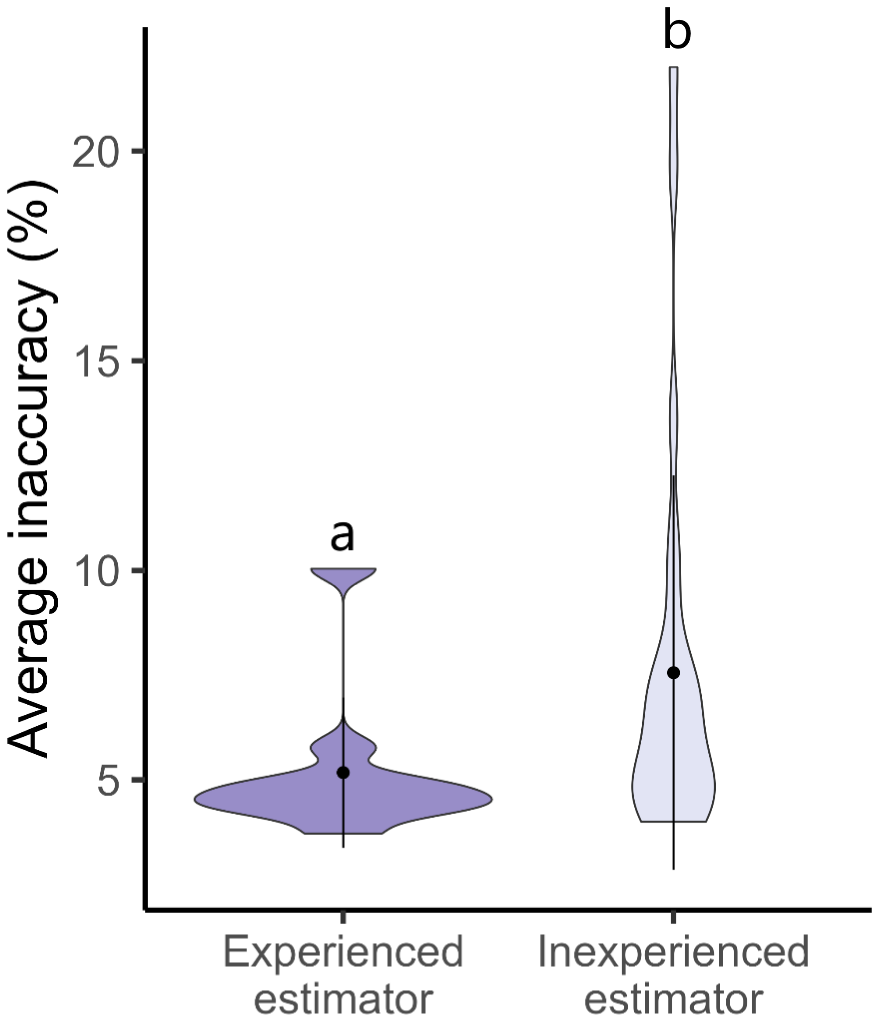
Violin plot comparing average baseline estimate inaccuracy between experienced and inexperienced cover estimators when measuring 50 images without feedback. Different letters above plots denote significant differences between groups (*p* = 0.047).

Participants with prior professional experience in estimating cover had significantly lower baseline estimate inaccuracy of 5.2% in comparison to inexperienced participants whose baseline inaccuracy averaged 7.6% (Figure 4; *p* = 0.047, *W* = 67).

## 4 Discussion

In just half an hour, the COVERater decreases users' inaccuracy of species cover estimates across both terrestrial and aquatic habitats from 7.6% before training to 5.2% after training (Figure 1). Our application is the first of its kind to improve the accuracy of cover estimates. We hope that our app will increase data quality in multiple fields, ranging from assessing the abundance of species, to facilitating more reliable data in biological monitoring programmes (e.g., the New Zealand National Vegetation Survey Databank and Australian Regional Land Partnerships Program). Most excitingly, the COVERater provides freely available standardised training for researchers worldwide, enabling more comparable data collection across global collaborative projects, and potentially increasing the scope and impact of future studies. A similar training tool used for estimating herbivory, the ZAX Herbivory Trainer (Xirocostas et al. 2022), has already been shared within or implemented across international collaborations such as the Herbivory Network (https://herbivory.lbhi.is/), the Bug Network (https://www.bug‐net.org/), the Herbivory Variability Network (https://herbvar.org/) and the Global Urban Evolution Project (https://www.globalurbanevolution.com/). We hope that the COVERater will follow a similar trajectory.

Participants who had experience in estimating per cent cover had lower levels of inaccuracy than inexperienced participants (Figure 4). These findings contrast with studies that found that previous experience does not impact the accuracy of visually estimating blood loss in hospitals (Dildy et al. 2004), substrate variables in streams (Wang, Simonson, and Lyons 1996) or fish length in reefs (Harvey, Fletcher, and Shortis 2001). However, when measuring tree diameter (Klein et al. 2022) or shark length (May et al. 2019), experienced estimators are more accurate than those with less experience. Thankfully, training with the COVERater reduces inexperienced participants' estimate inaccuracy to the same level as experienced estimators, enabling people of all research backgrounds to contribute robust scientific data to their field. Although we did not test the effectiveness of training on experienced estimators, we welcome and encourage them to also utilise our app.

We show that users' inaccuracy does not increase over time and remains consistent up to 135 days after initial training (Figure 3). To guarantee the quality of data arising from visual cover estimates, we conservatively suggest that scientists retrain with the COVERater every 3 months. We also recommend that users who have not completed the COVERater within 152 images or 64 min may be unsuitable to visually estimate cover, as they have exceeded the threshold where 90% of users have completed training (as in Xirocostas et al. 2022) (Figure 2A,B).

Citizen science projects are becoming increasingly popular to rapidly collect ecological data across greater areas (Kirchhoff et al. 2021; Moles and Xirocostas 2022) and over longer time frames (Gouraguine et al. 2019). The COVERater will improve the data quality of citizen scientists and provide an opportunity for professional scientists to expand the scope of their research to include larger volumes of publicly collected data, yielding greater power to answer big‐picture ecological questions (Moles and Xirocostas 2022; Wolf et al. 2022). However, it is important to note that not all observers should be expected to have ~5% accuracy even after training, as various factors in the field such as fatigue, more diverse/dense vegetation and variable light levels may impact estimates.

We now have globally available standardised training for visual estimates of cover (COVERater) and for herbivory (ZAX Herbivory Trainer) allowing for better data collection within and across projects. In the long run, such tasks will likely all be undertaken by artificial intelligence (AI), but for now, many of these tasks still require human input to ground truth outputs and calibrate or correct algorithms (Aitkenhead et al. 2003). For instance, a neural network trained to estimate lichen cover in boreal forests underperforms on images containing bright objects with high reflectance or uneven lighting (Lovitt et al. 2022). Until such time that AI accuracy in detecting species presence and estimating cover across a suite of ecosystems surpasses that of humans, our application provides the best method to ensure comprehensive and reliable data collection.

## Author Contributions

**Madelon M. Cruickshank:** data curation (lead), formal analysis (equal), investigation (lead), project administration (lead), writing – original draft (lead), writing – review and editing (supporting). **Angela T. Moles:** conceptualization (equal), formal analysis (equal), investigation (equal), supervision (equal), writing – original draft (supporting), writing – review and editing (supporting). **Samuel A. Debono:** data curation (supporting), software (lead), writing – review and editing (supporting). **Zoe A. Xirocostas:** conceptualization (equal), formal analysis (equal), investigation (equal), supervision (equal), visualization (lead), writing – original draft (supporting), writing – review and editing (lead).

## Ethics Statement

We received Human Research Ethics Advisory Panel approval (HC220176) from UNSW Sydney before commencing data collection.

## Conflicts of Interest

The authors declare no conflicts of interest.

## Data Availability

Code and data for all analyses are available on FigShare at this link (https://doi.org/10.6084/m9.figshare.23538855.v2). The COVERater can be found at https://coverater.com/.
